# Association of Previous COVID-19 Infection and Preoperative Haemoglobin with Transfusion Burden and Early Postoperative Outcomes Following Cardiac Surgery with Cardiopulmonary Bypass

**DOI:** 10.3390/jcm15176551

**Published:** 2026-08-25

**Authors:** Cornelia-Elena Predoi, Daniela Filipescu, Mihai Gabriel Stefan, Radu Filipescu, Dragos Guz, Cornelia Margineanu, Mihai Popescu, Cornel Robu, Serban-Ion Bubenek-Turconi, Niculae Iordache

**Affiliations:** 1 Emergency Institute of Cardiovascular Disease “Prof. Dr CC Iliescu”, 022322 Bucharest, Romania; corneliaelenapredoi@gmail.com (C.-E.P.); 1danielaf@gmail.com (D.F.); raduzfil@gmail.com (R.F.); guz.dragos@gmail.com (D.G.); cornelmd293@hotmail.com (C.R.); bubenek@alsys.ro (S.-I.B.-T.);; 2 Faculty of Medicine, University of Medicine and Pharmacy “Carol Davila”, 020021 Bucharest, Romania; margineanu.cornelia@gmail.com (C.M.); lcf4ever@yahoo.com (M.P.); 3 The University Emergency Hospital Bucharest, 050098 Bucharest, Romania; 4 Sf. Ioan Clinical Emergency Hospital, 042122 Bucharest, Romania

**Keywords:** COVID-19, cardiopulmonary bypass, anaemia, transfusion, cardiac surgery

## Abstract

**Background:** Preoperative anaemia and perioperative transfusion are associated with adverse outcomes after cardiac surgery with cardiopulmonary bypass (CPB). Coronavirus disease 2019 (COVID-19) may induce persistent haematological and endothelial alterations. Whether previous infection modifies the relationship between haemoglobin and outcomes is unclear. This study evaluated whether previous COVID-19 infection modified the association between preoperative haemoglobin concentration and perioperative transfusion burden after cardiac surgery with CPB. Early postoperative complications were analysed as predefined secondary outcomes. **Methods:** This study represents a secondary analysis of a prospective observational cohort of adult patients undergoing elective on-pump cardiac surgery between 1 August 2022 and 30 October 2023. Patients were categorised according to previous COVID-19 infection. Surgery was performed at least seven weeks after infection. The primary outcome was perioperative transfusion burden, defined as the total number of blood products administered from the intraoperative period until hospital discharge. An interaction term between previous COVID-19 and preoperative haemoglobin was included in multivariable linear regression. **Results:** A total of 280 patients were included, of whom 101 (36.1%) had a previous COVID-19 infection. Preoperative haemoglobin was comparable between patients with and without a previous COVID-19 infection (13.4 [12.4–14.8] vs. 13.8 [12.2–14.6] g/dL; *p* = 0.472). No significant differences in early postoperative complications, transfusion rates, or transfusion burden were observed according to previous COVID-19 infection. The COVID-19 × haemoglobin interaction was not statistically significant in the adjusted model (B = 0.002, 95% CI −0.081 to 0.085; *p* = 0.958). Lower preoperative haemoglobin, lower baseline platelet count, longer CPB duration, and CKD were independently associated with greater transfusion burden. In an exploratory multivariable analysis, preoperative anaemia remained associated with postoperative AKI after adjustment for relevant covariates (adjusted OR 5.80, 95% CI 2.23–15.06; *p* < 0.001). **Conclusions**: No statistically significant interaction between previous COVID-19 and preoperative haemoglobin was demonstrated for perioperative transfusion burden. Lower preoperative haemoglobin was independently associated with greater transfusion burden, while preoperative anaemia remained associated with postoperative AKI after multivariable adjustment in an exploratory analysis. These findings support systematic preoperative anaemia screening and Patient Blood Management in cardiac surgery.

## 1. Introduction

Cardiac surgery requiring cardiopulmonary bypass (CPB) remains associated with substantial perioperative morbidity despite continuous advances in surgical techniques, myocardial protection, and intensive care management [1,2]. Postoperative complications, including delirium, acute kidney injury (AKI), respiratory failure, low cardiac output syndrome, infections, and mortality, are strongly influenced by perioperative oxygen delivery and tissue perfusion [3,4,5,6]. Haemoglobin concentration plays a central role in this process by determining blood oxygen-carrying capacity and contributing directly to systemic oxygen transport during and after CPB.

Preoperative anaemia is highly prevalent among patients undergoing cardiac surgery, affecting approximately 20–50% of surgical candidates [7,8,9,10]. Anaemia and perioperative transfusion have both been identified as independent predictors of adverse outcomes in cardiac surgery, including prolonged intensive care stay, renal dysfunction, infection, and mortality [10,11]. In a large single-centre cohort of non-emergent cardiac surgery patients, performed in our centre, preoperative haemoglobin < 13 g/dL was strongly associated with increased transfusion requirements and longer hospitalisation [12]. Likewise, implementation of Patient Blood Management (PBM) strategies in cardiac surgery reduced transfusion exposure and perioperative complications, highlighting the importance of preoperative hematologic optimisation [13].

In the setting of CPB, anaemia may become particularly relevant because extracorporeal circulation induces haemodilution, haemolysis, systemic inflammation, endothelial activation, and microcirculatory dysfunction [14,15,16]. Consequently, relatively small reductions in preoperative haemoglobin concentration may impair oxygen delivery and have disproportionate clinical effects in patients undergoing CPB.

Since the emergence of Coronavirus disease 2019 (COVID-19), increasing evidence has demonstrated that SARS-CoV-2 infection may induce persistent haematologic and endothelial alterations extending beyond the acute phase of illness. Reported abnormalities include inflammatory anaemia, dysregulated iron metabolism characterised by hypoferremia, hyperferritinemia, increased hepcidin levels, reduced transferrin saturation, impaired erythrocyte deformability, and chronic endothelial dysfunction. Several of these alterations have been associated with impaired oxygen transport, persistent inflammation, and worse clinical outcomes following COVID-19 [17,18,19,20].

The optimal timing of elective surgery following COVID-19 has evolved throughout the pandemic and remains influenced by infection severity, persistence of symptoms, patient-related risk, and surgical complexity. Although several perioperative recommendations have used a minimum interval of seven weeks after SARS-CoV-2 infection, some studies have suggested that postoperative risk after major elective surgery may remain elevated for approximately eight weeks or longer [21,22]. In the present study, the interval of at least seven weeks represented a predefined eligibility criterion based on the perioperative recommendations applicable at the time of study design and was not intended to define the optimal timing of cardiac surgery after COVID-19. Uncertainty remains particularly relevant for high-risk procedures such as cardiac surgery requiring CPB. The inflammatory response induced by extracorporeal circulation may interact with residual endothelial dysfunction and hematologic abnormalities following SARS-CoV-2 infection, potentially influencing postoperative recovery. Previous COVID-19 infection has also been associated with persistent endothelial injury and extracellular matrix remodelling [18]. A recent systematic review including more than 38,000 patients demonstrated that surgery after COVID-19 was generally associated with postoperative outcomes comparable to baseline surgical risk, although evidence remains limited for high-risk surgical populations [23]. Likewise, our previous prospective study of patients undergoing elective cardiac surgery with CPB found no significant increase in vasoactive requirements or early postoperative complications among patients with previous COVID-19 infection more than seven weeks before surgery [24].

Whether previous COVID-19 infection modifies the relationship between preoperative haemoglobin concentration and perioperative transfusion requirements remains unknown. Persistent alterations in erythropoiesis, iron metabolism, endothelial function, and microcirculatory regulation may influence the physiological consequences of anaemia during CPB, even after apparent clinical recovery from COVID-19. Understanding this potential interaction may improve perioperative risk stratification and contribute to individualised PBM strategies in cardiac surgery.

Although previous studies, including our own prospective cohort and systematic review, have evaluated postoperative outcomes after delayed cardiac surgery following COVID-19 infection, none have specifically examined whether previous COVID-19 infection modifies the relationship between preoperative haemoglobin concentration and perioperative transfusion burden. We hypothesised that previous COVID-19 infection may modify the association between preoperative haemoglobin concentration and perioperative transfusion burden in patients undergoing cardiac surgery with CPB. Therefore, the primary aim of this study was to test this potential effect modification by evaluating the interaction between previous COVID-19 and preoperative haemoglobin concentration with respect to perioperative transfusion burden. Postoperative clinical outcomes and analyses stratified according to preoperative anaemia and COVID-19 were considered secondary exploratory analyses.

## 2. Materials and Methods

### 2.1. Study Design and Patient Population

This study represents a secondary analysis of a prospective observational cohort. The source cohort was prospectively established, and demographic, clinical, perioperative, and postoperative data were collected according to the predefined protocol of the original study [24]. All patients included in the original cohort who fulfilled the predefined eligibility criteria were included in the present secondary analysis. No additional inclusion or exclusion criteria were applied. The original cohort was established to investigate postoperative outcomes in patients with and without previous COVID-19. The study protocol prospectively specified that the collected data could be used for subsequent secondary analyses addressing additional research questions related to perioperative outcomes. The original prospective cohort recruited consecutive eligible patients between 1 August 2022 and 30 October 2023. No formal a priori sample-size calculation or predefined numerical recruitment target was established for the original cohort; its size was determined by the number of consecutive eligible patients enrolled during this predefined recruitment period. The original study was approved by the Institutional Ethics Committee (Approval No. 20083/11 July 2022). The study was prospectively registered at ClinicalTrials.gov (NCT05752162).

**Figure 1:**
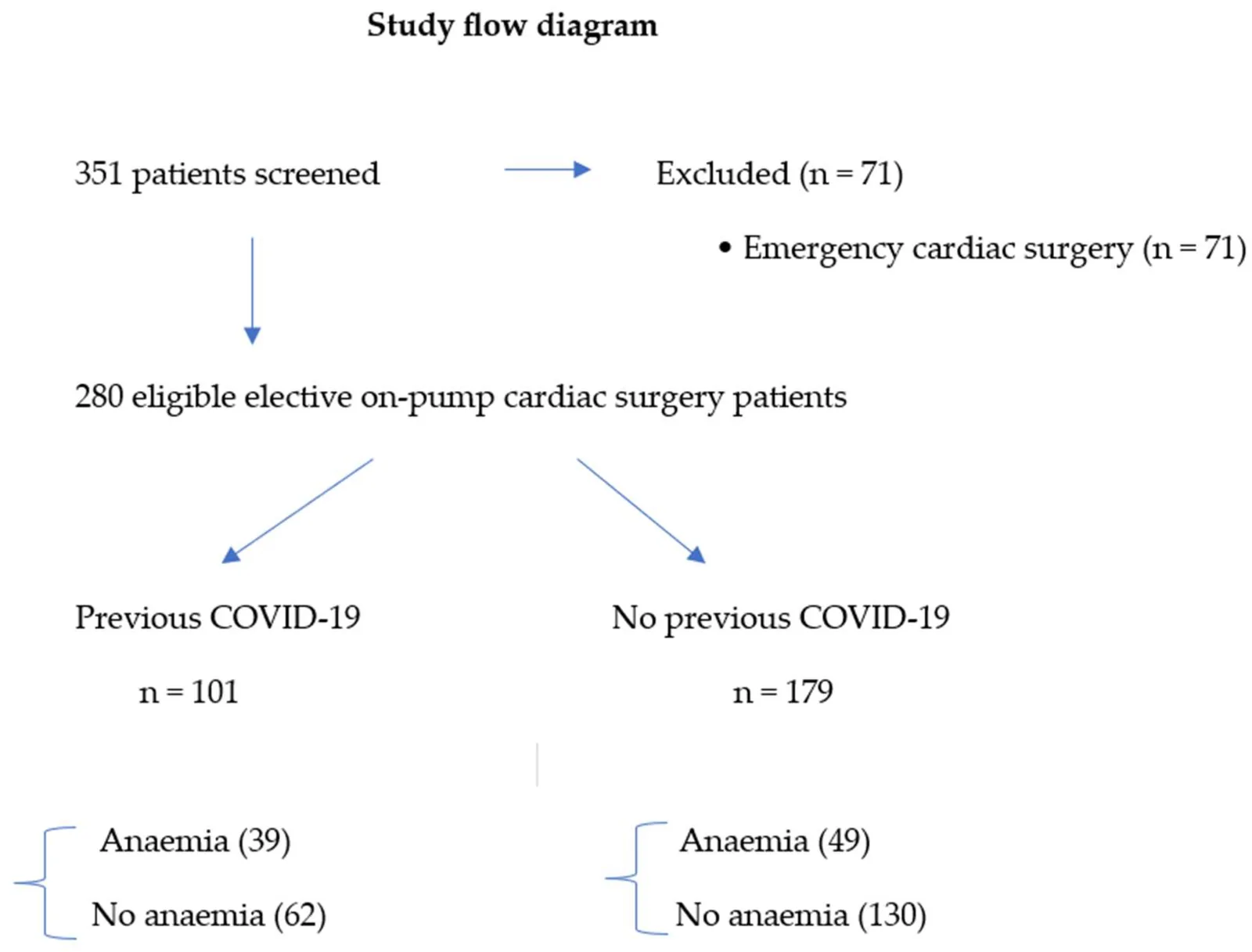
Flow diagram of patient selection for the study.

Consecutive adult patients (≥18 years) undergoing elective on-pump isolated coronary artery bypass grafting (CABG), valve surgery, or combined procedures were included. Patients with previous COVID-19 were eligible only if surgery was performed at least 7 weeks after SARS-CoV-2 infection, in accordance with contemporary perioperative recommendations and institutional practice [21]. Patients undergoing surgery within 7 weeks of infection were not eligible for inclusion. Based on the available clinical records, no patient included in the cohort required further postponement of surgery because of persistent COVID-19-related symptoms. Previous COVID-19 was defined as a documented positive SARS-CoV-2 polymerase chain reaction (PCR) or antigen test recorded in the patient’s medical records. Patients with documented SARS-CoV-2 infection occurring at least 7 weeks before cardiac surgery were classified as the “previous COVID-19” group, whereas patients without documented previous SARS-CoV-2 infection were classified as the “no previous COVID-19” group. These group definitions were used throughout the present analysis. Patients with active SARS-CoV-2 infection or with COVID-19 less than 7 weeks at the time of surgery, those undergoing emergency surgery, preoperative transfusion or off-pump cardiac surgery were excluded. No a priori sample-size calculation was performed for the present secondary analysis. The study size was determined by the number of eligible patients available in the original prospectively collected cohort. Therefore, the analysis was not specifically powered to detect an interaction between previous COVID-19 and preoperative haemoglobin concentration. A flow diagram summarising patient screening, eligibility, exclusions, and study inclusion is presented in Figure 1. The study is reported in accordance with the Strengthening the Reporting of Observational Studies in Epidemiology (STROBE) guidelines; the completed STROBE checklist is provided as Table S1 in the Supplementary Materials.

### 2.2. Data Collection

Demographic, clinical, perioperative, and postoperative data were prospectively collected according to the original study protocol. The variables included demographic characteristics, cardiovascular risk factors, preoperative clinical status, surgical variables, and postoperative outcomes, including AKI, pneumonia, acute respiratory failure, delirium, stroke, arrhythmias, acute cardiac failure, Intensive Care Unit (ICU) and hospital length of stay, vasoactive requirements, and in-hospital mortality [24].

Detailed information on baseline and in-hospital pharmacotherapy was not systematically collected as part of the original study protocol and was therefore not included in the present secondary analysis.

For the purposes of the present analysis, additional haematologic and transfusion-related variables were retrospectively extracted from institutional electronic medical records, laboratory databases, anaesthesia charts, and PBM documentation. The variables included preoperative hematologic parameters related to anaemia and iron metabolism, including haemoglobin concentration, ferritin, transferrin saturation, platelet count, and perioperative transfusion data. Preoperative anaemia was defined according to the World Health Organisation (WHO) guideline using sex-specific haemoglobin thresholds of <13 g/dL in men and <12 g/dL in women [25].

Ferritin and transferrin saturation were selectively measured in those with preoperative anaemia according to institutional PBM protocols. Consequently, these variables were available only for a subset of the study population.

Perioperative transfusion was evaluated as the administration of individual blood components (red blood cells—RBCs, fresh frozen plasma—FFP, and platelets). Platelet transfusion referred to pooled platelet concentrates according to institutional practice. Fresh frozen plasma was administered as standard adult units (approximately 250 mL per unit) according to institutional practice. Cryoprecipitate was not used at our institution during the study period. Perioperative transfusion decisions were made according to the institutional Patient Blood Management protocol in use throughout the study period. Red blood cell transfusion was generally considered at a haemoglobin concentration < 7.5 g/dL in haemodynamically stable patients, while at higher haemoglobin concentrations the decision was individualised according to active bleeding, haemodynamic instability, evidence of inadequate tissue oxygen delivery, and the overall clinical condition. Fresh frozen plasma was administered in the presence of clinically significant bleeding associated with documented or suspected coagulation factor deficiency, whereas platelet concentrates were administered for clinically significant bleeding associated with thrombocytopenia and/or impaired platelet function. Thus, transfusion decisions were based on both laboratory parameters and the clinical context rather than on isolated laboratory thresholds [26].

### 2.3. Outcomes

The primary objective of the present secondary analysis was to determine whether a previous COVID-19 infection modified the association between preoperative haemoglobin concentration and perioperative transfusion burden in patients undergoing elective cardiac surgery with cardiopulmonary bypass.

The primary outcome was perioperative transfusion burden, defined as the cumulative number of allogeneic blood products administered from the intraoperative period until hospital discharge, including RBC units, FFP units and platelet units. This composite outcome was selected to reflect the overall requirement for perioperative transfusion support rather than the administration of an individual blood product. No conversion or weighting between different blood products was applied; each transfused RBC unit, FFP unit, and platelet concentrate contributed one unit to the cumulative transfusion burden. Platelet transfusion referred to pooled platelet concentrates according to institutional practice. Secondary transfusion outcomes included the incidence of RBCs, FFP and platelets defined as the receipt of at least one unit of the respective blood product during the perioperative period.

Additional postoperative outcomes included AKI, acute respiratory failure, pneumonia, delirium, stroke, postoperative arrhythmias, acute cardiac failure, ICU length of stay, hospital length of stay, and in-hospital mortality. Acute kidney injury was defined according to the Kidney Disease Improving Global Outcomes (KDIGO) criteria [27]. Postoperative pneumonia was defined according to the Centres for Disease Control and Prevention/National Healthcare Safety Network (CDC/NHSN) surveillance criteria [28]. Delirium was assessed using the Confusion Assessment Method for the Intensive Care Unit (CAM-ICU) [29]. The remaining postoperative complications were diagnosed according to institutional clinical criteria.

The primary objective of the multivariable analysis was to determine whether previous COVID-19 modified the association between preoperative haemoglobin concentration and perioperative transfusion burden. Accordingly, the multivariable linear regression model included previous COVID-19, preoperative haemoglobin concentration, and their interaction term (COVID-19 × haemoglobin). The statistical significance of the interaction term was used to assess effect modification, whereas the coefficients for previous COVID-19 and preoperative haemoglobin represented the corresponding main effects. The multivariable model included previous COVID-19 history, preoperative haemoglobin concentration, the COVID-19 × haemoglobin interaction term, age, sex, body mass index, smoking status, diabetes mellitus, chronic kidney disease, baseline platelet count, CPB duration, and type of cardiac surgical procedure.

### 2.4. Statistical Analysis

Statistical analyses were performed using IBM SPSS Statistics version 26 (IBM Corp., Armonk, NY, USA). Continuous variables are presented as mean ± SD or median and IQR, as appropriate according to their distribution. Categorical variables are presented as number and percentage.

Continuous variables were compared using the independent samples Student’s *t*-test or the Mann–Whitney *U* test, as appropriate. Categorical variables were compared using the chi-square test or Fisher’s exact test when the expected cell count was less than five. For two-group comparisons of categorical secondary outcomes, effect estimates are additionally reported as risk ratios (RRs) with 95% confidence intervals.

For exploratory comparisons across the four groups defined by previous COVID-19 and preoperative anaemia status, categorical variables were compared using Pearson’s chi-square test or Fisher’s exact test, as appropriate, whereas continuous variables were compared using the Kruskal–Wallis test because of their non-normal distribution. For RBC transfusion analyses, transfusion rates were compared across the four groups using Pearson’s chi-square test, while the number of RBC units transfused was compared using the Kruskal–Wallis test among patients who received RBC transfusion. The reported *p*-values represent omnibus comparisons across the four groups. No post hoc pairwise comparisons were performed because these subgroup analyses were exploratory, no specific pairwise hypotheses were prespecified, and the number of patients and events within individual subgroups was limited. Accordingly, significant omnibus tests were interpreted as evidence of overall between-group differences without inference regarding specific pairwise comparisons. Because these analyses represented omnibus four-group comparisons and no pairwise contrasts were performed, pairwise effect estimates were not calculated.

Given the observed association between preoperative anaemia and postoperative AKI, an exploratory multivariable binary logistic regression analysis was performed to assess whether this association persisted after adjustment for baseline renal function and other clinically relevant covariates. Postoperative AKI was entered as the dependent variable, and preoperative anaemia, chronic kidney disease, previous COVID-19, age, sex, diabetes mellitus, and CPB duration were included as covariates. Adjusted associations were expressed as odds ratios (ORs) with 95% confidence intervals (CIs). A multivariable model for in-hospital mortality was not performed because only five deaths occurred in the cohort, resulting in an insufficient number of events for reliable multivariable adjustment.

The distribution of preoperative haemoglobin according to previous COVID-19 was illustrated using box-and-whisker plots.

Because the total number of blood products transfused contained a substantial proportion of zero values and demonstrated marked right-skewness, transfusion burden was calculated as the natural logarithm of (total blood products transfused + 1) before regression analysis. The addition of one allowed inclusion of patients with zero transfused blood products while reducing skewness and improving model fit.

Unadjusted linear regression analyses were initially performed to examine associations between candidate variables and perioperative transfusion burden. The multivariable model was specified based primarily on clinical relevance and potential confounding, rather than solely on statistical significance in the unadjusted analyses. To facilitate interpretation of the main effects in the interaction model and reduce non-essential multicollinearity, preoperative haemoglobin concentration was mean-centred at the cohort mean (13.435 g/dL) before construction of the interaction term. The interaction term was calculated as previous COVID-19 × mean-centred preoperative haemoglobin concentration. The multivariable model included previous COVID-19 history, mean-centred preoperative haemoglobin concentration, the COVID-19 × haemoglobin interaction term, age, sex, body mass index, smoking status, diabetes mellitus, chronic kidney disease, baseline platelet count, CPB duration, and type of cardiac surgical procedure. For interpretability, baseline platelet count was scaled so that the regression coefficient represents the change in transfusion burden per 10 × 10^9^/L increase in platelet count. Surgical procedure type was entered using dummy variables, with CABG as the reference category. Ferritin and transferrin saturation were not included in the primary multivariable model because these variables were available only in clinically selected subsets of patients according to institutional Patient Blood Management protocols. Because these variables were obtained selectively on the basis of clinical indication rather than by protocol for all patients, the resulting missing data were considered indication-driven and not suitable for population-level adjustment. Consequently, ferritin and transferrin saturation were not included in the primary multivariable regression model, which was performed using complete data from all 280 patients. Analyses involving ferritin and transferrin saturation were considered exploratory and are presented descriptively. Multicollinearity among the independent variables included in the multivariable model was assessed using variance inflation factors (VIFs).

All statistical tests were two-sided, and a *p*-value < 0.05 was considered statistically significant.

## 3. Results

A total of 351 patients were screened during the study period. After exclusion of 71 patients undergoing emergency cardiac surgery, 280 consecutive eligible patients undergoing elective on-pump cardiac surgery were included in the final analysis (Figure 1).

### 3.1. Baseline Characteristics

A total of 280 patients undergoing cardiac surgery with CPB were included, of whom 101 (36.1%) had a previous COVID-19 infection and 179 (63.9%) had no documented previous COVID-19 infection. Baseline demographic, clinical, haematological, and surgical characteristics of the study cohort are summarised in Table 1.

Patients with and without previous COVID-19 were generally comparable with respect to demographic and clinical characteristics, including age, body mass index (BMI), ASA status, NYHA functional class, EuroSCORE I, smoking status, chronic kidney disease, diabetes mellitus, and SARS-CoV-2 vaccination status, with no statistically significant between-group differences.

Male sex was more frequent among patients without previous COVID-19 compared with those with prior infection (69.8% vs. 57.4%, *p* = 0.049). Patients with previous COVID-19 also had a numerically higher prevalence of preoperative anaemia (38.6% vs. 27.4%), although this difference did not reach statistical significance (*p* = 0.061).

Regarding haematological parameters, preoperative haemoglobin concentration was comparable between groups, with median values of 13.4 g/dL [12.4–14.8] in patients with previous COVID-19 and 13.8 g/dL [12.2–14.6] in those without prior infection (*p* = 0.472).

Ferritin measurements were available in 30 patients and transferrin saturation in 40 patients. Among patients in whom iron studies were available, no significant differences were observed between groups.

Surgical complexity appeared comparable between groups, with no significant differences in CPB duration (1.55 [1.33–1.90] vs. 1.56 [1.26–1.98] hours, *p* = 0.946) or aortic cross-clamp duration (1.08 [0.88–1.36] vs. 1.10 [0.81–1.43] hours, *p* = 0.886), suggesting a similar intraoperative burden irrespective of previous COVID-19 infection. The distribution of cardiac surgical procedures was comparable between patients with and without previous COVID-19 (overall *p* = 0.743). Aortic valve replacement accounted for 26.7% versus 22.9%, CABG for 28.7% versus 29.6%, combined complex surgery for 28.7% versus 27.4%, mitral valve replacement for 8.9% versus 8.4%, and other procedures for 6.9% versus 11.7% of procedures in patients with and without previous COVID-19, respectively.

**Figure 2:**
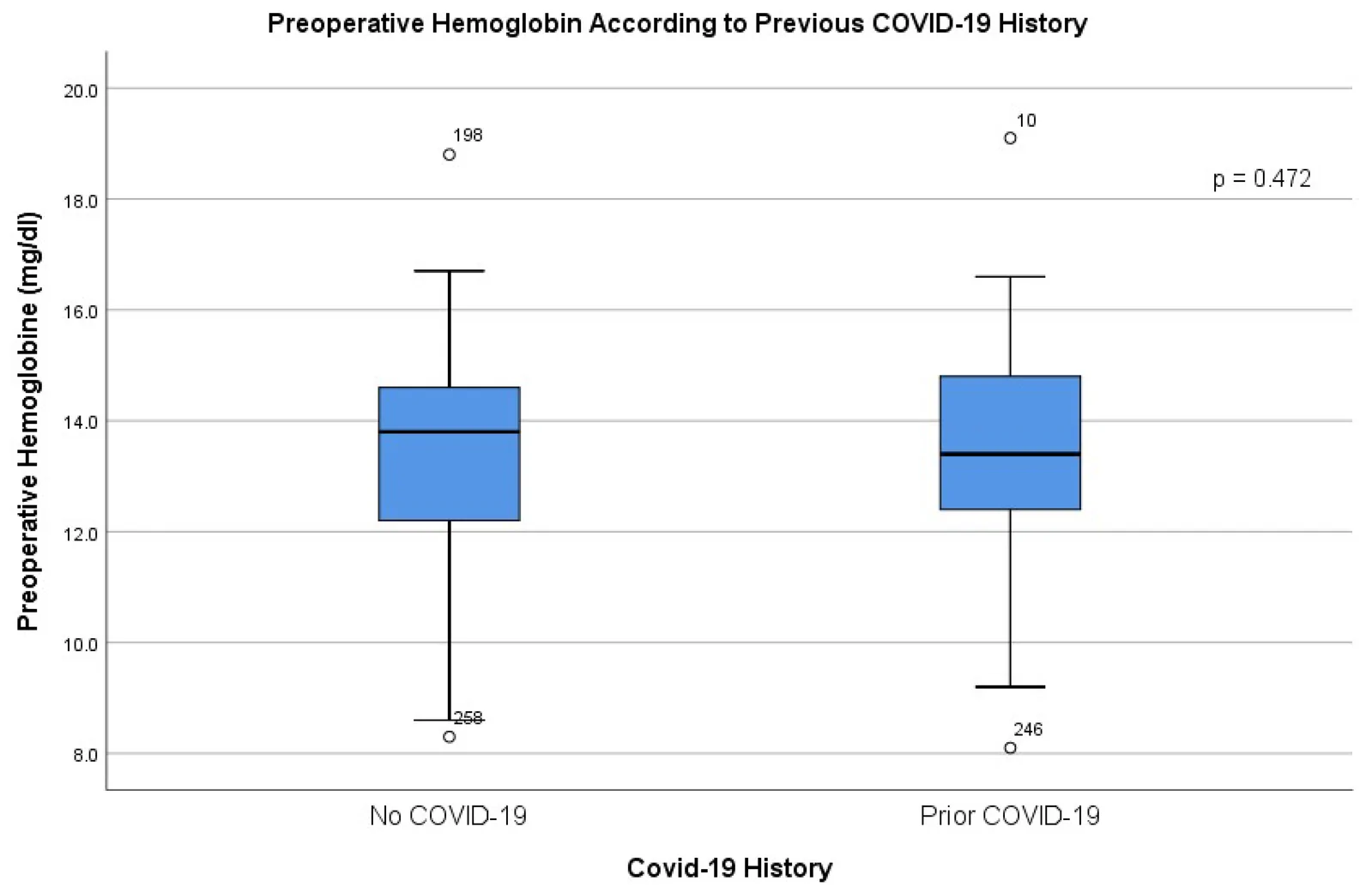
Preoperative haemoglobin distribution according to previous COVID-19. Boxes represent the interquartile range, horizontal lines indicate median values, whiskers denote the range excluding outliers, and circles represent patients with extreme values.

Preoperative haemoglobin concentrations were comparable between patients with and without previous COVID-19 infection (Figure 2), with no statistically significant difference between groups (median 13.4 vs. 13.8 g/dL, *p* = 0.472).

### 3.2. Postoperative Outcomes According to Preoperative Anaemia

Early postoperative outcomes according to preoperative anaemia status are presented in Table 2. Patients with preoperative anaemia experienced a significantly higher incidence of AKI compared with non-anaemic patients (22.7% vs. 4.7%, *p* < 0.001). In-hospital mortality was also significantly higher among anaemic patients (4.5% vs. 0.5%, *p* = 0.035).

In an exploratory multivariable logistic regression analysis, preoperative CKD was independently associated with postoperative AKI (adjusted OR 5.45, 95% CI 1.99–14.96; *p* = 0.001). Preoperative anaemia also remained associated with postoperative AKI after adjustment for CKD, previous COVID-19, age, sex, diabetes mellitus, and CPB duration (adjusted OR 5.80, 95% CI 2.23–15.06; *p* < 0.001). Longer CPB duration was additionally associated with postoperative AKI (adjusted OR 3.27 per hour, 95% CI 1.83–5.84; *p* < 0.001). Previous COVID-19, age, sex, and diabetes mellitus were not independently associated with postoperative AKI.

No statistically significant differences were observed between anaemic and non-anaemic patients regarding the incidence of delirium, stroke, acute respiratory failure, pneumonia, acute cardiac failure, or postoperative arrhythmias (all *p* > 0.05). Likewise, median hospital length of stay was comparable between the two groups (8 [7–10] vs. 8 [7–9] days, *p* = 0.531). Although patients with preoperative anaemia tended to have a longer ICU stay (median 3 [2–4] vs. 3 [2–3] days), this difference did not reach statistical significance (*p* = 0.083).

### 3.3. Exploratory Analysis According to Previous COVID-19 and Preoperative Anaemia

Early postoperative outcomes stratified according to both previous COVID-19 and preoperative anaemia status are presented in Table 3. Most postoperative complications, including delirium, stroke, acute respiratory failure, pneumonia, acute cardiac failure, and postoperative arrhythmias, did not show significant overall differences across the four groups (all *p* > 0.05).

The overall incidence of AKI differed significantly across the four groups (omnibus *p* < 0.001). Numerically, the highest incidence was observed in patients with preoperative anaemia and no previous COVID-19 infection (28.6%), followed by patients with both previous COVID-19 and preoperative anaemia (15.4%), whereas lower rates were observed in patients without anaemia (5.4% in the no-COVID-19/no-anaemia group and 3.2% in the COVID-19/no-anaemia group). ICU length of stay also differed overall across the four groups (omnibus *p* = 0.039). Because post hoc pairwise comparisons were not performed, these findings indicate overall between-group heterogeneity and should not be interpreted as evidence of statistically significant differences between specific pairs of groups. Hospital length of stay did not differ significantly across groups (*p* = 0.396).

In-hospital mortality was numerically highest in patients with preoperative anaemia and no previous COVID-19 infection (6.1%), followed by patients with both previous COVID-19 and anaemia (2.6%); however, the overall difference across the four groups did not reach statistical significance (*p* = 0.063).

### 3.4. Perioperative Blood Product Utilisation According to Preoperative Anaemia and Previous COVID-19

Perioperative blood product utilisation according to preoperative anaemia status is summarised in Table 4. Overall, patients with preoperative anaemia required RBC transfusion significantly more frequently than patients without anaemia (62.5% vs. 26.6%, *p* < 0.001). In contrast, the incidence of platelet transfusion (5.7% vs. 3.1%, *p* = 0.330) and FFP transfusion (6.8% vs. 5.2%, *p* = 0.578) did not differ significantly between the two groups. Patients with preoperative anaemia received RBC transfusion significantly more frequently than patients without anaemia (62.5% vs. 26.6%; RR 2.35, 95% CI 1.77–3.13; *p* < 0.001).

Perioperative transfusion rates according to previous COVID-19 are presented in Table 5. Overall, 106 patients (37.8%) received at least one RBC transfusion during the perioperative period. The incidence of RBC transfusion did not differ significantly between patients with and without previous COVID-19 (42.6% vs. 35.2%; RR 1.21, 95% CI 0.89–1.64; *p* = 0.249). Likewise, no statistically significant differences were observed in the administration of FFP (5.0% vs. 6.1%, *p* = 0.793). Platelet transfusion was numerically more frequent in patients with previous COVID-19 infection (6.9% vs. 2.2%), although the difference did not reach statistical significance (*p* = 0.062).

RBC transfusion rates differed significantly across the four groups (overall *p* < 0.001). Descriptively, RBC transfusion was more frequent among patients with preoperative anaemia, both in those without previous COVID-19 (63.3%) and those with previous COVID-19 (61.5%), compared with the corresponding groups without anaemia (24.6% and 30.6%, respectively). Among patients who received RBC transfusion, the number of RBC units transfused also differed across the four groups in the overall comparison (*p* = 0.026). Median RBC use was highest in the no COVID-19/anaemia group (2 [1–2] units), compared with 1 [1–1.5], 1 [1–1], and 1 [1–2] units in the other groups, respectively. As no post hoc pairwise comparisons were performed, these findings should be interpreted as exploratory overall group differences and do not establish statistically significant differences between individual pairs of groups (Table 6).

Complete data were available for the primary outcome and all covariates included in the primary multivariable model; therefore, the analysis included all 280 patients and no imputation was required. After multivariable adjustment for age, sex, body mass index, smoking status, diabetes mellitus, chronic kidney disease, baseline platelet count, CPB duration, and type of cardiac surgical procedure, lower preoperative haemoglobin concentration (B = −0.152, 95% CI −0.206 to −0.098; *p* < 0.001), lower baseline platelet count (B = −0.014 per 10 × 10^9^/L increase, 95% CI −0.025 to −0.002; *p* = 0.018), longer CPB duration (B = 0.275, 95% CI 0.167–0.384; *p* < 0.001), and chronic kidney disease (B = 0.361, 95% CI 0.158–0.564; *p* = 0.001) were independently associated with increased perioperative transfusion burden (Table 7). Compared with CABG, aortic valve replacement was associated with lower transfusion burden (B = −0.318, 95% CI −0.526 to −0.110; *p* = 0.003), while the other surgical procedure categories were not significantly associated with transfusion burden. At the mean preoperative haemoglobin concentration (13.435 g/dL), previous COVID-19 was not significantly associated with transfusion burden (B = 0.068, 95% CI −0.079 to 0.215; *p* = 0.360). No statistically significant interaction between previous COVID-19 and preoperative haemoglobin concentration was observed (B = 0.002, 95% CI −0.081 to 0.085; *p* = 0.958). No evidence of problematic multicollinearity was observed in the final model (all VIFs < 2).

Given the significant unadjusted association between preoperative anaemia and postoperative AKI, an exploratory multivariable logistic regression analysis was performed. After adjustment for chronic kidney disease, previous COVID-19, age, sex, diabetes mellitus, and CPB duration, preoperative anaemia remained associated with postoperative AKI (adjusted OR 5.80, 95% CI 2.23–15.06; *p* < 0.001). Preoperative chronic kidney disease (adjusted OR 5.45, 95% CI 1.99–14.96; *p* = 0.001) and longer CPB duration (adjusted OR 3.27 per hour, 95% CI 1.83–5.84; *p* < 0.001) were also associated with postoperative AKI after multivariable adjustment. No statistically significant adjusted associations with postoperative AKI were observed for previous COVID-19, age, sex, or diabetes mellitus.

In contrast, only five in-hospital deaths occurred in the cohort. Given the limited number of events, multivariable logistic regression for in-hospital mortality was not performed because it would have resulted in substantial overfitting and unstable effect estimates. Accordingly, the observed association between preoperative anaemia and in-hospital mortality should be considered unadjusted and exploratory.

## 4. Discussion

The principal contribution of the present study was to investigate whether previous COVID-19 modifies the association between preoperative haemoglobin concentration and perioperative transfusion burden in patients undergoing elective cardiac surgery with CPB. We found no evidence of a statistically significant interaction between previous COVID-19 and preoperative haemoglobin. The hypothesis underlying the present study was based on accumulating evidence that SARS-CoV-2 infection induces persistent haematological and inflammatory abnormalities extending beyond the acute phase of illness. Previous studies have described anaemia, thrombocytopenia, leukocyte abnormalities, endothelial dysfunction, dysregulated iron metabolism, and persistent inflammatory activation following COVID-19, all of which have the potential to impair oxygen delivery and tissue perfusion [17,20,23]. In a recent cross-sectional study, Nkansah et al. reported that more than 60% of hospitalised COVID-19 patients were anaemic, with thrombocytopenia and multiple leukocyte abnormalities also being highly prevalent, and these haematological alterations were associated with disease severity [30]. Similar disturbances in erythropoiesis, iron homeostasis, and inflammatory pathways have been consistently reported after SARS-CoV-2 infection, providing a biological rationale for investigating whether previous COVID-19 could influence the relationship between preoperative haemoglobin concentration and postoperative outcomes after cardiac surgery with CPB [17,18,19].

Despite this biological plausibility, we did not detect evidence of a statistically significant interaction between previous COVID-19 and preoperative haemoglobin with respect to perioperative transfusion burden. However, because interaction analyses generally require considerably larger sample sizes than analyses of main effects, our findings should not be interpreted as definitive evidence that such an interaction does not exist. Rather, they indicate that no interaction was demonstrated within the statistical power of the present study. Furthermore, previous COVID-19 was defined on the basis of documented SARS-CoV-2 infection. Patients with asymptomatic or undocumented infections may therefore have been misclassified into the no-previous-COVID-19-infection group. Such non-differential exposure misclassification would be expected to bias any true association or interaction toward the null, potentially reducing our ability to detect effect modification. Baseline haemoglobin concentration, platelet count, ferritin, transferrin saturation, and perioperative transfusion rates were comparable between patients with and without previous COVID-19 infection. Furthermore, the interaction term between previous COVID-19 and preoperative haemoglobin was not independently associated with transfusion burden in the multivariable model. These results suggest that, when elective cardiac surgery is performed at least seven weeks after SARS-CoV-2 infection, any residual haematological alterations are unlikely to be sufficiently pronounced to influence perioperative transfusion requirements or early postoperative outcomes.

Our findings are consistent with those of our previous prospective study, which demonstrated that patients undergoing elective cardiac surgery more than seven weeks after COVID-19 did not experience increased vasoactive requirements or a higher incidence of early postoperative complications compared with patients without previous infection [24]. However, the optimal interval between SARS-CoV-2 infection and major elective surgery remains uncertain. Deng et al. reported that postoperative risk varied according to the interval from infection to major elective surgery, with risk approaching baseline after longer recovery periods [22]. In the present cohort, the ≥7-week interval represented a predefined eligibility criterion based on recommendations applicable at the time of study design. Because patients undergoing earlier surgery were excluded and the exact date of SARS-CoV-2 infection was not available in the dataset used for this secondary analysis, the precise infection-to-surgery interval could not be evaluated. Heterogeneity in the time elapsed since infection may therefore have attenuated or masked potential time-dependent effects. Accordingly, our findings cannot establish whether seven weeks, eight weeks, or a longer interval represents the optimal timing for cardiac surgery with CPB.

Vaccination represents another relevant consideration when interpreting perioperative outcomes in patients with previous SARS-CoV-2 infection. In our cohort, vaccination status was recorded, although the study was not designed to evaluate vaccination as an effect modifier of the relationship between haemoglobin and transfusion burden. Strobel et al. specifically examined the effect of CPB on SARS-CoV-2 vaccine antibody levels, highlighting a potential interaction between extracorporeal circulation and vaccine-associated humoral immunity [31]. Because perioperative antibody titres were not measured in our cohort, the relationship between vaccination, humoral immunity, and CPB could not be evaluated. Further studies incorporating vaccination characteristics and measures of SARS-CoV-2-specific immunity may help clarify this relationship.

Exploratory subgroup analyses according to both previous COVID-19 and preoperative anaemia were broadly consistent with the primary analysis. Higher unadjusted rates of AKI and RBC transfusion were observed among patients with preoperative anaemia, whereas no clear pattern suggesting an adverse effect of previous COVID-19 was identified. Because these four-group comparisons were exploratory and no post hoc pairwise comparisons were performed, they should be interpreted cautiously.

Preoperative anaemia was associated with greater RBC transfusion requirements and higher rates of AKI in the present cohort, consistent with previous evidence identifying anaemia as an important perioperative risk factor in cardiac surgery [7,11,12,32]. Beyond haemoglobin concentration itself, preoperative iron status may also have prognostic relevance in cardiac surgery. Hazen et al. reported that abnormal iron status was common among patients undergoing elective cardiac surgery and was associated with an increased risk of postoperative major complications, although no significant association with RBC transfusion was observed [33]. In the present cohort, however, ferritin and transferrin saturation were measured only in clinically selected patients with suspected iron deficiency rather than systematically across the entire cohort. Consequently, these iron-related variables could not be reliably incorporated into the population-level multivariable model. Importantly, in the exploratory multivariable analysis performed in response to the observed association with AKI, preoperative anaemia remained associated with postoperative AKI after adjustment for chronic kidney disease, previous COVID-19, age, sex, diabetes mellitus, and CPB duration. Chronic kidney disease and longer CPB duration were also associated with AKI after multivariable adjustment, emphasising the multifactorial nature of postoperative renal injury. In contrast, although in-hospital mortality was higher among patients with preoperative anaemia in the unadjusted analysis, only five deaths occurred, precluding reliable multivariable adjustment. The association between preoperative anaemia and mortality should therefore be considered exploratory and should not be interpreted as evidence of an independent association. These anaemia-related findings primarily provide clinical context rather than constitute the novel contribution of this analysis. The distinctive objective of the present study was to determine whether previous COVID-19 modified the established relationship between preoperative haemoglobin concentration and perioperative transfusion burden. Although the primary outcome combined different blood products with distinct clinical indications, it was intended to represent overall perioperative transfusion support rather than biological equivalence between transfusion components. Accordingly, individual blood products, particularly RBC transfusion, were also analysed separately to provide additional descriptive information regarding the components contributing to overall perioperative transfusion support.

In the multivariable model, lower preoperative haemoglobin concentration, lower baseline platelet count, longer CPB duration, and chronic kidney disease were independently associated with increased transfusion burden, whereas previous COVID-19 and the COVID-19 × haemoglobin interaction were not. Overall, the present findings extend previous work by specifically examining the potential modifying effect of previous COVID-19 on the established relationship between preoperative haemoglobin and perioperative transfusion requirements.

### Limitations

This study has several limitations. First, no a priori sample-size calculation was performed specifically for the COVID-19 × haemoglobin interaction. As the present study represents a secondary analysis of an existing prospectively collected cohort, the sample size was determined by the number of eligible patients available in the original cohort. Although the interaction between previous COVID-19 and preoperative haemoglobin concentration was prespecified as the primary research question of this secondary analysis, the study was therefore not specifically powered to detect interaction effects. Detecting statistical interactions generally requires larger sample sizes than detecting main effects. The 95% confidence interval around the interaction coefficient (B = −0.002, 95% CI −0.085 to 0.082) indicates that the data remain compatible with interaction effects of approximately ±0.08 on the log-transfusion scale per 1 g/dL difference in preoperative haemoglobin. Thus, although a large interaction was not observed, smaller effect-modification effects cannot be excluded. Consequently, the absence of a statistically significant interaction should be interpreted as a failure to demonstrate effect modification in this cohort rather than evidence of its absence, and smaller interaction effects cannot be excluded.

Second, previous COVID-19 was defined according to documented SARS-CoV-2 infection. Consequently, patients with asymptomatic or undocumented infections may have been misclassified into the no-previous COVID-19 group. Such non-differential exposure misclassification would be expected to bias any true association or interaction towards the null, potentially reducing our ability to detect effect modification. Third, although the original cohort was prospectively assembled, the present study represents a secondary analysis, and several haematological and transfusion-related variables were retrospectively extracted from institutional medical records.

Fourth, ferritin and transferrin saturation were not routinely measured in all patients but only in those with clinically suspected iron deficiency according to institutional Patient Blood Management protocols. Consequently, analyses involving iron-related parameters were based on a limited subset of patients and should be considered exploratory. Fifth, the study was conducted at a single tertiary cardiovascular centre, which may limit the generalisability of the findings to institutions with different patient populations, surgical case mix, transfusion practices, and Patient Blood Management protocols. In addition, the exploratory multivariable analysis of postoperative AKI included seven covariates despite only 29 AKI events. The relatively low number of events in relation to the number of model parameters increases the risk of overfitting and may limit the stability and precision of the adjusted estimates. Accordingly, the results of this analysis should be interpreted as exploratory and require confirmation in larger cohorts.

Sixth, the number of in-hospital deaths was low, limiting the precision of mortality estimates; therefore, the observed association between preoperative anaemia and mortality should be interpreted cautiously. In addition, multiple secondary and subgroup analyses were performed without adjustment for multiple comparisons. These analyses were exploratory; therefore, the possibility of inflated type I error and false-positive findings should be considered when interpreting nominally significant results. The composite transfusion burden combined different blood products with distinct clinical indications and dosing characteristics. Although this measure was selected to reflect overall perioperative transfusion exposure, it does not imply biological equivalence between RBCs, plasma and platelets, and should therefore be interpreted as a global measure of transfusion support rather than a standardised transfusion dose. In addition, although log-transformation was used to address the right-skewed distribution and substantial proportion of zero values in the composite transfusion outcome, alternative generalised count models, such as negative binomial, hurdle, or zero-inflated models, were not evaluated. The use of these alternative modelling approaches could potentially yield different effect estimates and should be considered in future studies. Furthermore, several established determinants of perioperative transfusion, including redo surgery, left ventricular function, nadir haematocrit during CPB, detailed baseline and in-hospital pharmacotherapy, including perioperative antiplatelet and anticoagulant therapy, and blood conservation techniques, were not systematically available for adjustment. Residual confounding therefore cannot be excluded.

An important limitation is the heterogeneity in the interval between previous COVID-19 and cardiac surgery. Although all patients in the previous COVID-19 group underwent surgery at least 7 weeks after documented SARS-CoV-2 infection, the exact date of infection was not retained in the dataset used for this secondary analysis. Only the year of infection was available; therefore, the precise infection-to-surgery interval could not be calculated, and patients could not be reliably stratified into clinically meaningful post-infection time intervals. Categorisation based solely on calendar year was considered insufficiently precise because it could result in substantial misclassification of the actual time elapsed since infection. Consequently, potential time-dependent effects of previous COVID-19 could not be evaluated. Given that residual haematological, inflammatory, and endothelial abnormalities may vary with time after SARS-CoV-2 infection, heterogeneity in the infection-to-surgery interval may have attenuated or masked an association between previous COVID-19 and perioperative outcomes, including a potential interaction with preoperative haemoglobin concentration.

## 5. Conclusions

In patients undergoing elective cardiac surgery with CPB at least seven weeks after documented SARS-CoV-2 infection, we observed no significant differences in early postoperative complications, transfusion rates, or perioperative transfusion burden according to previous COVID-19. In this cohort, we found no evidence of a statistically significant interaction between previous COVID-19 and preoperative haemoglobin concentration with respect to perioperative transfusion burden. However, because the study was not specifically powered to detect interaction effects, smaller or time-dependent modifications of this relationship cannot be excluded. Lower preoperative haemoglobin concentration was independently associated with greater perioperative transfusion burden, while preoperative anaemia remained associated with postoperative AKI after multivariable adjustment in an exploratory analysis. Although in-hospital mortality was higher among patients with preoperative anaemia in the unadjusted analysis, the limited number of deaths precluded reliable multivariable adjustment, and this finding should therefore be interpreted cautiously. These findings support systematic preoperative anaemia screening and optimisation within Patient Blood Management programmes in patients undergoing cardiac surgery with CPB.

## Figures and Tables

**Table 1:** Baseline demographic, clinical, haematological, and surgical characteristics according to previous COVID-19 infection.

Patients’ Characteristics	All Cohort (*n* = 280 Patients)	Previous COVID-19 (*n* = 101 Patients)	No Previous COVID-19 (*n* = 179 Patients)	*p*-Value
**Demographics**				
Age (years)—median [IQR]	65 [55–69]	66 [54–69]	64 [55–69]	0.939
Male gender (*n*, %)	183 (65.4)	58 (57.4)	125 (69.8)	0.049
BMI preoperatively—kg/m^2^ (mean ± SD)	27.8 ± 4.9	27.7 ± 4.8	27.9 ± 5	0.752
**Clinical**				
ASA—median [IQR]	3 [3–4]	3 [3–4]	3 [3–5]	0.284
NYHA—median [IQR]	3 [2–3]	3 [2–3]	3 [2–3]	0.072
EuroSCORE I—median [IQR]	5 [3–6]	5 [3–6]	4 [3–6]	0.269
Smoking (*n*, %)	137 (48.9)	46 (45.5)	91 (50.8)	0.455
CKD *—(*n*, %)	43 (15.4)	14 (13.9)	29 (16.2)	0.730
Diabetes mellitus (*n*, %)	80 (28.6)	29 (28.7)	51 (28.5)	1.000
SARS-CoV-2 Vaccination (*n*, %)	173 (61.8)	64 (63.4)	109 (60.8)	0.703
**Haematological variables**				
Anaemia * (*n*, %)	88 (31.4)	39 (38.6)	49 (27.4)	0.061
Haemoglobin (g/dL) median [IQR]	13.6 [12.2–14.6]	13.4 [12.4–14.8]	13.8 [12.2–14.6]	0.472
Ferritin ng/mL median [IQR]	211 [44.8–380.3] (n = 30)	206 [87.7–300.2] (n = 10)	271.6 [42.1–464.9] (n = 20)	1.000
Transferrin saturation % median [IQR]	16.2 [10.6–21.7] (n = 40)	13.9 [10–19.1] (n = 14)	16.4 [10.9–22.1] (n = 26)	0.685
Platelets/microL median [IQR]	231,000 [196,000–281,000]	219,000 [188,500–278,500]	238,000 [202,000–282,000]	0.059
**Surgical variables**				
**Type of cardiac surgery, *n* (%)**				0.743
Aortic valve replacement	68 (24.3)	27 (26.7)	41 (22.9)	
CABG	82 (29.3)	29 (28.7)	53 (29.6)	
Combined complex surgery	78 (27.9)	29 (28.7)	49 (27.4)	
Mitral valve replacement	24 (8.6)	9 (8.9)	14 (8.4)	
Other surgery	28 (10)	7 (6.9)	21 (11.7)	
CPB duration (hours)—median [IQR]	1.55 [1.27–1.96]	1.55 [1.33–1.90]	1.56 [1.26–1.98]	0.946
Aortic clamping duration (hours)—median [IQR]	1.1 [0.85–1.41]	1.08 [0.88–1.36]	1.1 [0.81–1.43]	0.886

* Anaemia defined according to WHO criteria (<13 g/dL in men and <12 g/dL in women); CKD = estimated glomerular filtration rate < 60 mL/min/1.73 m^2^; caccination: ≥1 dose of any WHO-authorised COVID-19 vaccine before surgery; CABG = coronary artery bypass grafting; CPB = cardiopulmonary bypass; CKD = chronic kidney disease; IQR—interquartile range.

**Table 2:** Association between preoperative anaemia and postoperative outcomes following cardiac surgery with cardiopulmonary bypass.

Patients’ Outcomes	Overall Cohort (*n* = 280)	Anaemic Patients (*n* = 88)	Patients Without Anaemia (*n* = 192)	RR (95% CI)	*p*-Value
Delirium (*n*, %)	18 (6.4)	7 (8)	11 (5.7)	1.39 (0.56–3.46)	0.600
Stroke (*n*, %)	3 (1.1)	1 (1.1)	2 (1)	1.09 (0.10–11.87)	1.000
Acute respiratory failure (*n*, %)	65 (23.2)	21 (23.9)	44 (22.9)	1.04 (0.66–1.64)	0.880
Pneumonia (*n*, %)	9 (3.2)	4 (4.5)	5 (2.6)	1.75 (0.48–6.34)	0.469
Acute cardiac failure (*n*, %)	30 (10.7)	12 (13.6)	18 (9.4)	1.45 (0.73–2.89)	0.302
Arrhythmias (*n*, %)	65 (23.2)	20 (22.7)	45 (23.4)	0.97 (0.61–1.54)	1.000
AKI (*n*, %)	29 (10.4)	20 (22.7)	9 (4.7)	4.85 (2.30–10.21)	<0.001
Hospitalisation LOS—median [IQR]	8 [7–10]	8 [7–10]	8 [7–9]	-	0.531
ICU LOS—median [IQR]	3 [2–3]	3 [2–4]	3 [2–3]	-	0.083
Mortality (*n*, %)	5 (1.8)	4 (4.5)	1 (0.5)	8.73 (0.99–76.96)	0.035

Definitions: delirium, positive Confusion Assessment Method for the Intensive Care Unit (CAM-ICU) assessment; stroke, new focal neurological deficit lasting >24 h with imaging confirmation; acute respiratory failure, reintubation or requirement for non-invasive ventilation with PaO_2_/FiO_2_ < 300 mmHg; pneumonia, diagnosed according to Centres for Disease Control and Prevention/National Healthcare Safety Network (CDC/NHSN) criteria; acute cardiac failure, low cardiac output syndrome requiring inotropic or mechanical circulatory support; arrhythmias, new atrial fibrillation/flutter or sustained ventricular arrhythmias requiring treatment; AKI, defined according to Kidney Disease Improving Global Outcomes (KDIGO) criteria; LOS—length of stay.

**Table 3:** Postoperative outcomes according to combined previous COVID-19 and preoperative anaemia.

Patients’ Outcomes	Overall Cohort (*n* = 280)	No COVID-19/No Anaemia (*n* = 130)	No COVID-19/ Anaemia (*n* = 49)	COVID-19/No Anaemia (*n* = 62)	COVID-19/ Anaemia (*n* = 39)	*p*-Value
Delirium (*n*, %)	18 (6.4)	7 (5.4)	5 (10.2)	4 (6.5)	2 (5.1)	0.681
Stroke (*n*, %)	3 (1.1)	2 (1.5)	1 (2)	0 (0)	0 (0)	0.616
Acute respiratory failure (*n*, %)	65 (23.2)	30 (23.1)	15 (30.6)	14 (22.6)	6 (15.4)	0.414
Pneumonia (*n*, %)	9 (3.2)	4 (3.1)	2 (4.1)	1 (1.6)	2 (5.1)	0.778
Acute cardiac failure (*n*, %)	30 (10.7)	11 (8.5)	8 (16.3)	7 (11.3)	4 (10.3)	0.506
Arrhythmias (*n*, %)	65 (23.2)	30 (23.1)	10 (20.4)	15 (24.2)	10 (25.6)	0.944
AKI—(*n*, %)	29 (10.4)	7 (5.4)	14 (28.6)	2 (3.2)	6 (15.4)	<0.001
Hospitalisation LOS—median [IQR]	8 [7–10]	8 [7–10]	8 [7–11]	8 [7–9]	8 [7–10]	0.396
ICU LOS—median [IQR]	3 [2–3]	3 [2–3]	3 [2–4]	3 [2–3]	3 [2–4]	0.039
Mortality (*n*, %)	5 (1.8)	1 (0.8)	3 (6.1)	0 (0)	1 (2.6)	0.063

*p*-values represent overall (omnibus) comparisons across the four groups. No post hoc pairwise comparisons were performed because these analyses were exploratory; therefore, pairwise effect estimates and confidence intervals are not reported. Definitions: delirium, positive Confusion Assessment Method for the Intensive Care Unit (CAM-ICU) assessment; stroke, new focal neurological deficit lasting >24 h with imaging confirmation; acute respiratory failure, reintubation or requirement for non-invasive ventilation with PaO_2_/FiO_2_ < 300 mmHg; pneumonia, diagnosed according to Centres for Disease Control and Prevention/National Healthcare Safety Network (CDC/NHSN) criteria; acute cardiac failure, low cardiac output syndrome requiring inotropic or mechanical circulatory support; arrhythmias, new atrial fibrillation/flutter or sustained ventricular arrhythmias requiring treatment; AKI, defined according to Kidney Disease Improving Global Outcomes (KDIGO) criteria, LOS—length of stay.

**Table 4:** Association between preoperative anaemia and perioperative blood product utilisation.

Variable	All Cohort (*n* = 280)	Anaemic Patients (*n* = 88)	Patients Without Anaemia (*n* = 192)	RR (95%CI)	*p*-Value
RBC transfusion, *n* (%)	106 (37.8)	55 (62.5)	51 (26.6)	2.35 (1.77–3.13)	<0.001
Platelet transfusion, *n* (%)	11 (3.9)	5 (5.7)	6 (3.1)	1.82 (0.57–5.80)	0.330
FFP transfusion, *n* (%)	16 (5.7)	6 (6.8)	10 (5.2)	1.31 (0.49–3.49)	0.578

*n* = number of patients; RRs compare anaemic with non-anaemic patients; patients without preoperative anaemia were used as the reference group.

**Table 5:** Perioperative transfusion rates according to previous COVID-19.

Variable	All Cohort (*n* = 280)	Previous COVID-19 (*n* = 101)	No Previous COVID-19 (*n* = 179)	RR (95% CI)	*p*-Value
RBC transfusion, *n* (%)	106 (37.8)	43 (42.6)	63 (35.2)	1.21 (0.89–1.64)	0.249
Platelet transfusion, *n* (%)	11 (3.9)	7 (6.9)	4 (2.2)	3.10 (0.93–10.34)	0.062
FFP transfusion, *n* (%)	16 (5.7)	5 (5)	11 (6.1)	0.81 (0.29–2.25)	0.793

*n* = number of patients; RRs compare patients with previous COVID-19 with those without previous COVID-19; patients without previous COVID-19 were used as the reference group.

**Table 6:** Perioperative red blood cell transfusion according to previous COVID-19 and preoperative anaemia.

Group	RBC, *n* (%)	Median RBC Units Transfused Among Transfused Patients [IQR]
No COVID-19/No anaemia (*n* = 130)	32 (24.6)	1 [1–1.5]
No COVID-19/Anaemia (*n* = 49)	31 (63.3)	2 [1–2]
COVID-19/No anaemia (*n* = 62)	19 (30.6)	1 [1–1]
COVID-19/Anaemia (*n* = 39)	24 (61.5)	1 [1–2]
Overall *p*-value	<0.001	0.026

*n* = number of patients; *p*-values represent overall comparisons across the four groups. RBC transfusion rates were compared using Pearson’s chi-square test or Fisher’s exact test, as appropriate, and RBC units among transfused patients using the Kruskal–Wallis test. No post hoc pairwise comparisons were performed.

**Table 7:** Univariable and multivariable linear regression analysis of factors associated with transfusion burden following cardiac surgery with cardiopulmonary bypass.

Variables (log_transfusion Dependent Variable)	Univariate		Multivariate		VIF
	B Coefficient (95% CI)	*p*-Value	B Coefficient (95% CI)	*p*-Value	
Previous COVID-19 (yes)	0.107 (−0.065, 0.279)	0.223	0.068 (−0.079, 0.215)	0.360	1.042
CPB duration (hours)	0.337 (0.214,0.461)	<0.001	0.275 (0.167, 0.384)	<0.001	1.117
Age (years)	0.000 (−0.008, 0.007)	0.910	−0.004 (−0.010, 0.003)	0.234	1.122
Male gender (yes)	−0.288 (−0.459, −0.117)	0.001	−0.160 (−0.331, 0.010)	0.066	1.380
Smoking (yes)	−0.090 (−0.255, 0.076)	0.287	0.013 (−0.137, 0.162)	0.868	1.164
BMI preoperatively—kg/m^2^	−0.012 (−0.029, 0.005)	0.153	−0.002 (−0.017, 0.012)	0.779	1.089
Diabetes mellitus (yes)	0.037 (−0.146, 0.220)	0.690	−0.009 (−0.156, 0.175)	0.910	1.164
Preoperative haemoglobin (g/dL)	−0.161(−0.205, −0.118)	<0.001	−0.152 (−0.206, −0.098)	<0.001	1.867
Baseline platelet count (per 10 × 10^9^/L increase)	−0.008 (−0.020, 0.005)	0.236	−0.014 (−0.025,−0.002)	0.018	1.146
Interaction term (previous COVID-19 × preoperative haemoglobin)	0.003 (−0.009, 0.016)	0.600	−0.002 (−0.081,0.085)	0.958	1.603
CKD (yes)	0.387 (0.162, 0.612)	0.001	0.361 (0.158, 0.564)	0.001	1.124
AVR vs. CABG	−0.153 (−0.375, 0.068)	0.174	−0.318 (−0.526, −0.110)	0.003	1.663
Combined complex surgery vs. CABG	0.245 (0.031, 0.458)	0.025	−0.013 (−0.212, 0.186)	0.897	1.668
MVR vs. CABG	−0.272 (−0.585, 0.041)	0.089	−0.314 (−0.602, −0.025)	0.033	1.369
Other surgery vs. CABG	0.005 (−0.290, 0.301)	0.973	−0.114 (−0.387, 0.160)	0.414	1.411

The multivariable analysis included all 280 patients. R^2^ = 0.337; adjusted R^2^ = 0.299. Preoperative haemoglobin was mean-centred at the cohort mean (13.435 g/dL) before construction of the interaction term. Accordingly, the coefficient for previous COVID-19 represents its estimated association with transfusion burden at the mean preoperative haemoglobin concentration. Baseline platelet count was scaled so that its coefficient represents the change in transfusion burden per 10 × 10^9^/L increase. CABG was used as the reference category for surgical procedure type. B = unstandardised regression coefficient; CI = confidence interval; CABG = coronary artery bypass grafting; AVR = aortic valve replacement; MVR = mitral valve replacement; CPB = cardiopulmonary bypass; CKD = chronic kidney disease.

## Data Availability

The data are not publicly available due to ethical restrictions involving patient confidentiality, but are available from the corresponding author upon reasonable request.

## Supplementary Materials

The following supporting information can be downloaded at: https://www.mdpi.com/article/10.3390/jcm15176551/s1, Table S1. STROBE Statement—checklist of items that should be included in reports of observational studies.

## Abbreviations

The following abbreviations are used in this manuscript:

ICU	Intensive Care Unit
SD	Standard Deviation
IQR	Interquartile range
CPB	Cardiopulmonary bypass
RBC	Red blood cell
FFP	Fresh frozen plasma
AKI	Acute kidney injury
PBM	Patient Blood Management
CABG	Coronary artery bypass grafting
CI	Confidence interval
